# Neural encoding of large-scale three-dimensional space—properties and constraints

**DOI:** 10.3389/fpsyg.2015.00927

**Published:** 2015-07-14

**Authors:** Kate J. Jeffery, Jonathan J. Wilson, Giulio Casali, Robin M. Hayman

**Affiliations:** ^1^Institute of Behavioural Neuroscience, Research Department of Experimental Psychology, Division of Psychology and Language Sciences, University College London, London, UK; ^2^Clinical and Experimental Epilepsy, Institute of Neurology, Faculty of Brain Sciences, University College London, London, UK

**Keywords:** spatial cognition, navigation, place cells, grid cells, head direction cells, theoretical model, dimensions

## Abstract

How the brain represents represent large-scale, navigable space has been the topic of intensive investigation for several decades, resulting in the discovery that neurons in a complex network of cortical and subcortical brain regions co-operatively encode distance, direction, place, movement etc. using a variety of different sensory inputs. However, such studies have mainly been conducted in simple laboratory settings in which animals explore small, two-dimensional (i.e., flat) arenas. The real world, by contrast, is complex and three dimensional with hills, valleys, tunnels, branches, and—for species that can swim or fly—large volumetric spaces. Adding an additional dimension to space adds coding challenges, a primary reason for which is that several basic geometric properties are different in three dimensions. This article will explore the consequences of these challenges for the establishment of a functional three-dimensional metric map of space, one of which is that the brains of some species might have evolved to reduce the dimensionality of the representational space and thus sidestep some of these problems.

## Introduction

The neural encoding of large-scale, navigable space has been one of the most intensively studied cognitive domains of the past several decades, ever since the seminal (and recently Nobel-prizewinning) discovery of place cells by O’Keefe and Dostrovsky (1971). Most of this work has involved rodents, and has resulted in elucidation of a set of interconnected brain structures whose function seems to be (at least partly) to assemble, from incoming sensory information, a representation of current location and heading direction. This representation appears to be used not only in self-localization and navigation, but also in memory. Recently, attention has been turning to the question of how the spatial encoding processes, hitherto studied in two dimensions, might operate in three. The third dimension adds considerable complexity to the computational problem and the purpose of this article is to explain why this is so, focusing on the underlying geometrical constraints operating in three-dimensional space that are not present in two. We advance a proposal for how this complexity might be accommodated, at least for surface-dwelling animals, which is that the cognitive representation of space, or “cognitive map,” is reduced to a mosaic of two-dimensional maps. We suggest that rather than being fully volumetric, the cognitive map is “multi-planar,” thus simplifying the encoding problem and sidestepping some of the problems that otherwise arise.

### A Brief Word about Nomenclature in Three Dimensions

Before proceeding, we begin with a brief note about representing three-dimensional space and the relevant terminology, to clarify the analysis that follows. A specific terminology is used to describe position, orientation and movement in 3D space. The fixed parts of the space from which the parameters are measured comprise, collectively, the *frame of reference*. The term *dimension* refers to the number of parameters needed to specify a point in a space. For example, an animal running back and forth on a linear track (a long thin structure, like a balance beam) might be considered to be moving in a one-dimensional space (if one disregards small lateral movements and the turning around at the track ends)—its position can thus be described by a single number selected from a continuous range (e.g., usually distance along the track) and its orientation by one of two categorical values (e.g., running left vs right, or running East vs West). The reference frame for this depiction is provided by the track itself. An animal exploring an open-field arena is moving in two dimensions and its position now needs two numbers to describe it, one from the x dimension and one from the y dimension, and its orientation is now also selected from a continuous range (e.g., 0–360°, or degrees East of North etc.). The reference frame is defined by the arena and if it is rectilinear (as in many laboratory studies) then the two orthogonal axes are naturally the edges of the apparatus, and the orientation relative to some fixed reference point (usually the top of the camera view).

Once an animal moves into the third dimension and starts to explore vertical space, the picture becomes more complex. Position is now described by three continuous variables, x and y, as before, plus z (height)—and orientation by three as well, which would typically be orientation in the x-y plane (also often called *azimuth*), angle in the vertical plane (*pitch*), and orientation around the long axis (*roll*).

**FIGURE 1:**
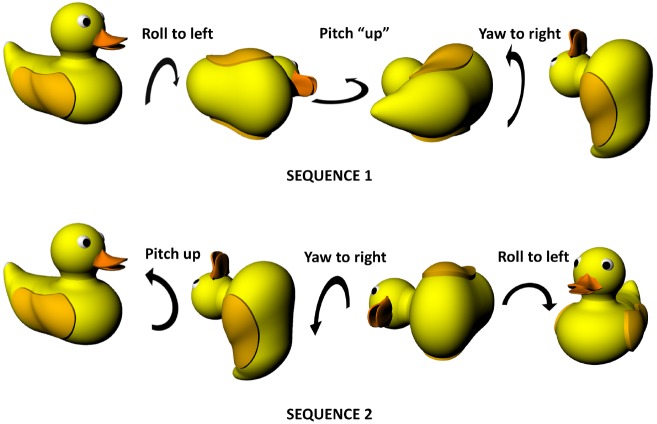
**Non-commutativity of rotations in three dimensions, showing that the same set of rotations (a pitch, a roll and a yaw) result in differing outcomes depending on the order in which they occur.** Note that the rotations are expressed in an egocentric reference frame (from the point of view of the duck)—the same effect holds true for rotations expressed in allocentric co-ordinates (relative to the earth). Duck model adapted with permission from a design by Ozan Kayíkcí (https://grabcad.com/library/duck-4).

As well as static position, it is necessary to be able to describe movements in three dimensions. There are two kinds of movement in these dimensions, linear and angular. For angular movements, i.e., rotation, the picture is complicated by the ensuing angular dissociation of reference frames, such that rotations need to be described either in terms of the planes referenced to the earth, or the planes referenced to the animal. In practical terms, it is easier to use the animal-centered (*egocentric*) frame. The following description is visualized in Figure 1. Rotation of an animal in the plane of its feet (rotating the animal around to face to the left or the right) is called *yaw*; rotation in the vertical plane orthogonal to this (i.e., the rotation that tips the nose—or, in this example, beak—up and down) is called *pitch* and rotation in the other vertical plane, the coronal plane, which tips the ears up and down, is called *roll*. Pitch is sometimes also called *elevation*, but since this term also often applies to static linear height above ground, we will use pitch here.

Figure 1 illustrates an important property of rotations in three dimensions which is that the order in which they occur is important, unlike two-dimensional rotations which can occur in any order with the same outcome. This is because rotation occurs on a plane and—since a plane is a two-dimensional entity—must at minimum involve two dimensions. Since there are only three spatial dimensions altogether, this means that rotations occurring in orthogonal planes must share one of their dimensions—they cannot be completely independent in the way that linear motion can be. Specifically, what happens is that rotation in one plane alters the axis of rotation in another, with consequences for how successive rotations unfold. This non-independence of rotations is why they become order-dependent (non-commutative). Also (or perhaps another way of looking at the same issue), it is the case that successive rotations in two orthogonal planes can result in a rotation having occurred in the third plane as well. As is discussed below, this has implications for how a stable compass signal could be maintained in a three-dimensional, volumetric space, which in turn impacts upon the other components of self-localisation such as position estimation.

How does the brain encode position and heading of its owner? A number of discoveries in recent decades have revealed the existence of a system of spatially sensitive neurons that respond to (“encode”) position, orientation or distance traveled, which we will briefly describe (see also Marozzi and Jeffery, 2012); for a more detailed review see (Moser et al., 2008). We begin with neurons encoding position, the place cells, followed by those encoding facing direction, the head direction cells (Sharp et al., 2001; Taube, 2007), and conclude with the most-recently discovered distance-sensitive neurons, the grid cells, which have begun to reveal the intrinsically metric nature of spatial encoding.

## Place Cells

**FIGURE 2:**
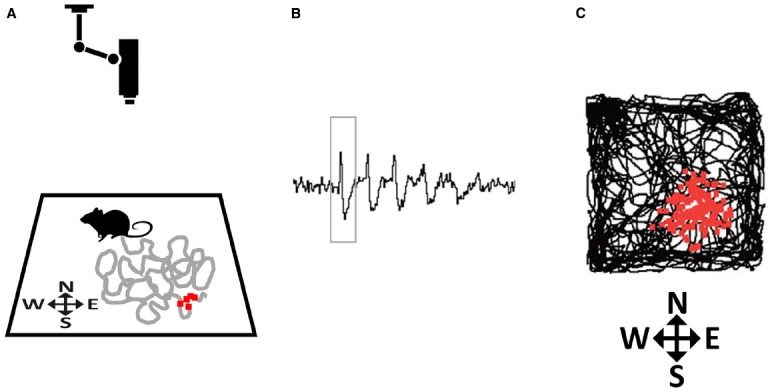
**How place fields are recorded. (A)** A rat explores a small environment (in this case, a square arena) while being tracked by an overhead camera. The gray line tracks the recent path of the rat and the small red squares depict action potentials (spikes) emitted by a hippocampal neuron that was recorded from implanted microelectrodes, plotted at the point where they occurred. **(B)** Spikes (one of which is outlined by the rectangle) as they appear on an oscilloscope screen. **(C)** At the end of the trial the complete set of accumulated spikes (small red squares) are overlaid on the total path of the rat (black line), and reveal a spatial clustering, in this case toward the South-East corner of the arena.

The first type of spatially modulated neurons to be discovered was reported by O’Keefe and Dostrovsky (1971), who found cells in the CA1 pyramidal layer of the hippocampus whose firing dramatically increased every time an exploring animal traversed one particular region of its environment (O’Keefe and Dostrovsky, 1971). They coined the term *place cells* for these cells, and defined the *place field* as the discrete region of space where each place cell would fire (Figure 2). The authors suggested that, by encoding the animal’s location, place cells at a population level function as a neural map.

Much work in the decades since place cells were discovered has revealed that the cells are sensitive not only to constellations of sensory stimuli in the environment, but also to abstract metric spatial quantities such as direction faced and distance traveled (see Jeffery, 2007, for review). Particularly important for place field localization in rats and mice, and possibly other species, are the boundaries of the immediate space (e.g., the walls of a room) which play an important part in determining where a place cell will fire—moving boundaries, for example, will tend to result in corresponding movement of the place field position (O’Keefe and Burgess, 1996). How these abstract quantities—distance, direction, boundedness etc.—are conveyed to place cells became apparent following the discovery of head direction cells (conveying direction) and grid cells (conveying distance), as discussed later.

Do place cells encode position in three-dimensional space or do they only encode position on a plane? We first examine what would, in principle, be observed if place cells form a fully volumetric map, and then review the sparse body of experimental data to date that speak to this issue.

### Place Cells in Three Dimensions—Theoretical Considerations

A place field is something of an abstract entity—it is the region in space encompassed by the spatially coherent firing of a place cell (i.e., not including random out-of-field spiking). In thinking about whether place cells encode volumetric space, the question thus is whether, if animals could move freely through the volume surrounding them, the region in which the cell spiked would have three-dimensional structure, or whether the cell would only fire when the animal neared or was actually *on* the floor. In the latter case we would consider the place field map to be only a two-dimensional map of the environment surface, and in the former case it would be a true, three-dimensional map.

**FIGURE 3:**
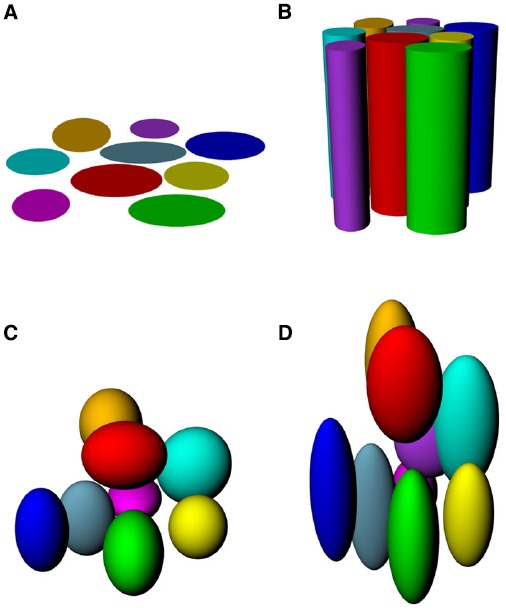
**Hypothetical structure of place fields in volumetric space. (A)** Place fields might be “flat” and not extend into the vertical dimension at all; **(B)** Place fields might extend into the vertical dimension, but only show spatial structure in the horizontal plane; **(C)** Place fields might be globular, extending into all three dimensions and being distributed evenly throughout the volume of space; **(D)** Place fields might be volumetric, but asymmetric (different scale in vertical than horizontal dimensions).

If place fields are not simply flat (Figure 3A) but extend into 3D space, then there are two obvious possibilities for how they might do this (Figures 3B–D). One is that they may be “cylindrical,” being ovoid in the horizontal plane but maintaining this shape at all heights, being essentially insensitive to height (Figure 3B), and the second is that they might be “globular,” maintaining their ovoid shape in all three dimensions and having equal metric properties (size and shape) in all three dimensions (Figure 3C). As a variant on this, they may be globular but with different metric properties for vertical space than they are for horizontal, being, say, elongated or compressed in vertical space relative to horizontal space (Figure 3D).

One more issue to consider is that of the species under consideration: the metric properties of place fields in different species may differ depending on their ecological niches. Do whales and dolphins form the same kinds of spatial representations that rats do? This seems *a priori* unlikely, given what we know about the importance of boundaries in establishing place field location—on the other hand, it may be that place cells function for self-localization only in specific situations (for example being in small enclosed spaces) and do so the same way in all mammals, regardless of their particular ecological niche, but are just more useful for some species than others. Cross-species comparative studies will be needed to answer this question.

We turn now to the experimental literature to see if it can shed light on the underlying likely structure of place fields in three-dimensional space.

### Place Cells in Three Dimensions—Data

The first study to investigate this issue experimentally was carried out by Knierim and McNaughton (2001), who recorded place cells from animals running on a rectangular linear track and compared the firing patterns observed on it to those displayed by the same cells after tilting portions of the track by 45°. If place fields had remained aligned to the horizontal plane (for example, as in Figure 3B) then they should have looked more elongated on the sloping section, while if place field intrinsic underlying structure is globular, occupying a fixed position in volumetric space (like Figures 3C,D) then they should have increased or decreased in size as the sloping track transected the “globules” at different levels. In fact, while place cells sometimes responded to the change in slope by changing their firing locations (“remapping”), they generally appeared to simply remain attached to the (now sloping) surface, possibly due to the salience of the available cues on the flat portions of the track.

More recently, Hayman et al. (2011) recorded place cells in a foraging task where rats were trained to climb on projecting pegs embedded onto a vertical board (the “pegboard”). It was found that place cells showed a significant increase in the length of both the major and minor axes of the fields, as well as an increase in the aspect ratio (major/minor axis ratio), compared to recordings of the same cells on a flat arena. These analyses suggested an impaired capacity by place cells to accurately represent the vertical dimension, encoding it with lower resolution (consistent with Figure 3D), but nevertheless still encoding it (i.e., the fields were not columnar as in Figure 3C). Combined with a similar finding on a different 3D apparatus (a vertical helix) together with an even more pronounced observation of field-elongation in grid cells (detailed below), these authors proposed that volumetric space is encoded anisotropically: that is, differently in vertical vs horizontal dimensions.

While the above experiments suggest that place fields are not inherently globular, it may be that the planar nature of the apparatus in these studies constrained the behavior of the fields. To determine if this is so it would be necessary to record place cells as animals move freely in a volumetric space. Such experiments have recently been reported in bats by Yartsev and Ulanovsky (2013). Bats are an interesting species for the study of three-dimensional spatial encoding because they, unlike rats (or indeed almost any other mammal) are able to move in three-dimensional environments without the constraint of needing a supporting substrate on which to move. These authors recorded place cells in these animals flying in large rooms by using a wireless neural-telemetry system (Yartsev and Ulanovsky, 2013). They found, in contrast to the results in rats discussed above, that place fields in the flying bats were isotropic, having the same size/shape in all dimensions, and lacking the elongation seen in the pegboard experiment of Hayman et al. (2011). This may mean that the species differ in their spatial encoding due to their different evolutionary histories: however, it could also be due to the difference in life experience of the subjects, or the difference in navigational constraints operating on a vertical plane vs. a 3D volume—further experiments will be needed to distinguish these possibilities.

The distribution of place fields in volumetric space provides some clues as to the structure of the underlying neural map of space, but does not tell us whether this is truly metric, even in bats. For this, we need to look at cells known to encode the explicitly metric properties of distance and direction. With this in mind we turn next to the head direction cells, to see what would be required for a compass system to be able to encode direction in all three dimensions, and then to examine data speaking to this issue.

## Head Direction Cells

**FIGURE 4:**
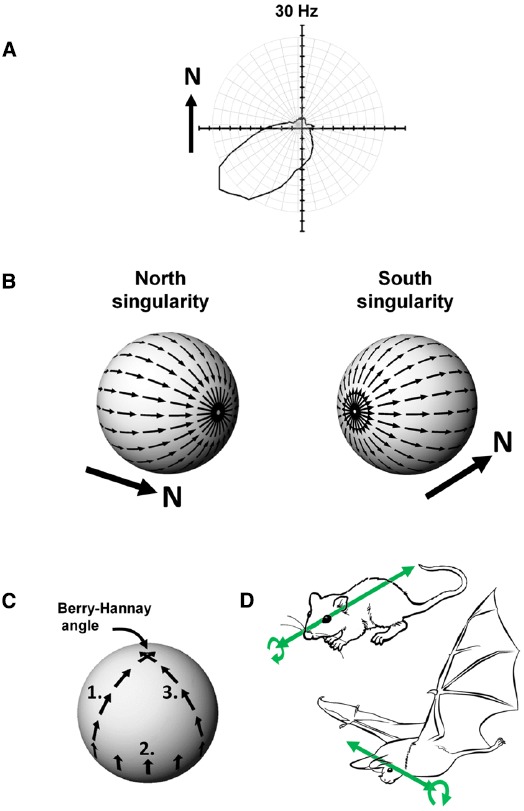
**(A)** Firing of a head direction cell, expressed as a polar plot of head direction (in the Earth-horizontal plane) vs. firing rate, showing a preference for spiking when the animal faces South-West. **(B)** The Hairy Ball problem: it is not possible to coat the surface of a sphere with smoothly changing vectors because there are always two singularities. In this example, a “North”-indicating head direction cell would have to abruptly reverse its firing direction (with respect to the sphere) when the animal crosses the Northernmost or Southernmost poles of the sphere. **(C)** Acquisition of a rotational error, the Berry-Hannay angle, when a notional “North”-facing head direction cell is transported around the surface of a sphere. In this example, first the animal undergoes a pitch rotation in moving from the top of the sphere to the equator (1), followed by a roll rotation as the animal sidles (still facing upward) around the equator by 90° (2), and then another pitch rotation as the animal climbs back to the top of the sphere (3). Although the animal never made a yaw rotation at any stage, the head direction cell has rotated its preferred firing direction by 90° counter-clockwise. **(D)** Possible differences in directional reference frames between rats and bats. In rats, which roll a lot (curved arrow) but pitch rarely, the stable reference for head direction cells may be the antero-posterior axis (the long axis of the head; straight arrow). A given head direction cell will thus be unaffected by rolls. In bats, which pitch a lot but roll rarely, the stable reference may be the transverse axis (across the ears) with the head direction cell being unaffected by pitch changes.

Head direction cells were discovered in rat cortex by Ranck in the 1980s (Ranck, 1984) and have been further characterized through numerous experiments over the intervening years by Taube, beginning with the seminal description of these cells in two papers co-published with Ranck in 1990 (Taube et al., 1990a,b). Any given head direction cell exhibits maximal spiking when an animal faces a given direction within the environment (Figure 4A), and as a population these cells can thus represent any given direction of a rat’s head within the horizontal plane. The direction in which a given cell spikes is known as its “preferred firing direction.”

Head direction cells derive their directional tuning from a variety of internal and external inputs. External inputs come primarily from visual cues in the environment (Goodridge et al., 1998) and are important for maintaining a stable directional signal—that is, a consistent preferred firing direction. While external inputs anchor the head direction signal to the outside world, inputs from self-motion signals update the signal as the animal turns its head through space. The internal sources for directional tuning include angular velocity input from the semicircular canals of the vestibular system, translational velocity signals from the otolith organs of the vestibular system, and may also include proprioceptive and motor efference signals; they are internal in the sense that they arise from movements of the animal’s own body through space, irrespective of external environmental landmarks. The input from the combined directional signals acts to move activity smoothly from one cell to the next as an animal turns its head through space—this smooth movement is thought to be orchestrated by the internal connections of the circuit which form a so-called attractor network, in which activity arises from the combined effect of afferent inputs and internal network dynamics (Skaggs et al., 1995; Zhang, 1996).

### Head Direction Cells in Three Dimensions

If an animal is moving over a three-dimensional surface and trying to maintain a constant sense of allocentric (world-centered) direction, then the head direction system has a complicated set of computations to perform. We present here two important problems that they face. The first, known as the hairy-ball problem, concerns an obstacle to configuring the attractor processes discussed above if an animal moves over a surface in three dimensional space. The second is responsible for the subtle introduction of errors in the head direction signal and arises from a phenomenon known as the Berry-Hannay angle.

#### Theoretical Considerations I—The Hairy Ball Problem

Imagine an animal is walking over a spherical surface, like a hill, and trying to maintain its sense of direction. What do its head direction cells do? We can think of the firing direction of each HD cell as a vector. However, a problem arises when one tries to smoothly carpet a spherical surface with vectors, a simple example being trying to comb hair flat on a round head; the hairy ball theorem, proved by Brouwer (1912), states that it is impossible to do this without creating a “crown” (in the case of hair) or a “singularity” (for a mathematician)—a place where all the heads or all the tails of the vectors run into each other (Figure 4B). To see how this applies to head direction cells, imagine the rat walking on the sphere. If one thinks of head direction cell activity as a vector, with direction being the preferred firing direction of the cell and length being the firing rate, then clearly the cells face a hairy ball problem: there will be a place on the sphere where the vectors run into each other head-to-head or tail-to-tail, and activity has to jump “across” the attractor from a given cell to one whose firing direction is completely opposed (i.e., on the other side of the “ring”). An animal in this position would be faced with having to “flip” its active head direction cell from (say) a North-indicating one to a South-indicating one. It is not clear how an attractor network could generate such discontinuous jumps, and as we will see below, it may be that nature has dealt with this problem by, as it were, hiding the singularity in almost-never-visited behavioral space.

#### Theoretical Considerations II—The Berry-Hannay-angle Problem

The second problem for head direction cells on a sphere arises because of a property of physical systems first described for quantum mechanics by Berry (1984) and then extended to classical systems by Hannay (1985). Berry noted that transport of a spinning particle around the surface of a sphere would cause the particle to have acquired a change in its spin direction by the time it returned to its starting point, even though it was never explicitly rotated: the rotation of the particle emerges from the fact that the surface of the sphere is itself “rotated” in 3D space. In Berry’s words, this property of acquired rotation, known as anholonomy, can be described in a simple thought experiment:

Take a pencil, lay it on the north pole of a globe and point it in the direction of any of the meridians: the lines of longitude that radiate from the pole. Move the pencil down along the line to the equator and, keeping it perpendicular to the equator, slide it to another line of longitude. Move the pencil back to the north pole along the new meridian, and you will find that although the pencil has been returned to its starting point and at no time was rotated, it no longer points along the original line of longitude. (Berry, 1988)

This effect on parallel transport of vectors over a curved surface is shown in Figure 4C. Now, if we replace “pencil” with “head direction cell firing,” we can see that if a head direction cell fires, say, when a rat faces upward as it sidles its way around the equator of a sphere, that when the rat walks back toward the top of the sphere the cell’s firing direction relative to the azimuth would be different depending on the animal’s starting point on the equator. Since the animal has not at any point made a yaw rotation (the rotation to which head direction cells are sensitive) then the firing of the cells would not have had the opportunity to take this hidden rotation into account, and would thus have acquired a rotation known as a Hannay (or more properly Berry-Hannay) angle.

An experiment with place cells in three dimensions during space flight suggests the Berry-Hannay angle problem can be surmounted by the spatial system, at least under conditions of low gravity (Knierim et al., 2000). In an experiment conducted during the Neurolab Space Shuttle mission, rats circumnavigated a three-arm “Escher” maze that was twisted through 3D space so that with three right-angle turns, the rat was back at its starting point. Although in the horizontal plane three right-angle rotations would lead to a rotational offset of 90° relative to the starting direction, place cells did not precess around the track, suggesting that the system was able to detect and compensate for the unusual geometry, perhaps by using distal visual cues and/or local olfactory ones.

At present, we do not know if head direction cells can avoid acquiring Berry-Hannay-angle errors, but it is interesting to speculate about what would be required for them to do so. Essentially, the head direction system would need to detect that the surface on which the animal was moving did itself rotate through three dimensions, and it would need to update the directional firing of the neurons to account for this. Thus, when the animal in the equatorial excursion scenario discussed above arrived back at its starting point, the acquired rotation would have been detected, and used to update the head direction signal appropriately.

Below, we look at some of the current data concerning the operation of head direction cells as animals move off the horizontal plane and begin to explore 3D space.

### Head Direction Cells in Three Dimensions—Data

Although rats do not move freely through volumetric space, and also do not move around comfortably on a sphere, they can be induced with some bribery to climb a vertical surface, and even—with utmost reluctance—to walk upside down for a short amount of time. This has enabled elucidation of some of the properties of the cells as rats venture away from the horizontal plane, and have provided some insights as to what the cells do as the surface the rat walks on (the locomotor surface) changes its orientation in 3D space. In addition to the discussion below, the reader is referred to the review by Taube (2011).

One of the first studies of head direction cells in a vertical environment, by Stackman et al. (2000) showed that as animals move from a horizontal surface to a vertically oriented wall, head direction cells maintain their directional firing such that the preferred firing direction of each cell on the floor is directly translated onto the wall, as if the wall were simply an extension of the floor. For example, a cell that is firing as the animal moves toward a vertical wall will continue firing as the animal moves onto the wall even though it now faces upward. The authors suggested that the space of possible firing directions in three dimensions would form a hemi-torus, which is the shape that would emerge if the ordinary 2D tuning curve of the cell were rotated between +/–90° pitch. A hemi-torus thus predicts how the cell should fire for a given head direction at a given head pitch (although, having only 2 degrees of freedom, it does not take into account roll).

Taube et al. (2013) subsequently showed that when rats climb in a vertical plane, head direction cells remain modulated by yaw rotations of the animal’s head occurring in that plane, just as they do when moving in a horizontal planar environment. Interestingly, these authors also found that when animals moved onto the vertical plane from different directions the cells’ firing directions relative to the vertical plane were systematically different for the differently oriented planes, so as to maintain consistency with the firing direction on the floor. However, this occurred only when the rat locomoted of its own volition and thus had a full set of self-motion cues to help with spatial updating; in cases when the animal was placed passively onto the wall then the cells adopted a local reference frame (for example, always firing when the rat faced upward, regardless of the wall orientation). And finally, Calton and Taube (2005) made the observation that when rats walk upside-down, there is a degradation of directional firing, whereby 47% of cells lose all directional selectivity, while others exhibit broader tuning curves with an increased background firing rate; such degradation in firing may explain why the animals experience difficulty with map-based spatial navigation while inverted (Valerio et al., 2010; Gibson et al., 2013). We will return to why this is interesting shortly, after having looked at the case for bats.

Finkelstein et al. (2015) have recently reported a study of head direction cells in bats; these cells have much lower firing rates than their rodent counterparts but still show clear directional tuning. Bats walking over a tilted or curved surface were found to have several groups of head direction cells modulated by yaw, pitch or roll independently, and some cells which conjunctively encoded for two of these (e.g., yaw and pitch); in some cases single cells were modulated by all three cardinal rotations. Since such cells have not been reported in rats (other than cells that are modestly modulated by pitch), it is possible that bats have evolved an enhanced capacity to represent three-dimensional space.

One of the most surprising findings from the Finkelstein et al study was what happened when the bats turned upside-down. Unlike the general degradation of directional firing in the inverted rats of Calton and Taube (2005) those head direction cells in the bats that maintained directional firing (about 40%) showed an unexpected *reversal* of firing direction, such that a cell that fired when the animal’s head pointed (say) North in the upright posture would fire when the head faced South in the inverted posture. In other words, if a bat were to walk from the floor to the wall to the ceiling of its cave, a given head direction cell would keep firing even though the animal had completely reversed its head direction by the end of its journey. These authors also, like Stackman et al. (2000) recruited a toroidal model to describe the activity of the neurons—although, as with the Stackman et al. (2000) hemi-torus, this model does not account for the entire movement space of the animals (it neglects roll) and so is not a complete description of head direction activity in all possible angles and postures.

This curious behavior—reversal of firing during inversion—seems mystifying at first (what use could such cells be for navigating?) until one considers that although the head direction as specified by the nose has reversed, another head-centered reference, the interaural axis—the line through the ears—has *not* reversed. During the bat’s movement from floor to ceiling, the left ear always continues to point (say) West, and the right ear to point East, regardless of what the nose is doing. Thus, if the cells are referenced to the ears instead of to the nose (Figure 4D) their firing can now be seen as directionally invariant, and would therefore be entirely useful for navigation. Thus, it could be said that while rats have head direction cells, bats have “ear direction” cells. The information needed for navigation is still present and correct, just referenced differently.

Why this difference between bats and rats? An appealing possibility has to do with the natural movements of the two species—bats frequently pitch 180° from upright to inverted, as when, for example, they swoop in to land on the ceiling, while they roll to inversion much less frequently (Finkelstein et al., 2015). The nose thus changes direction far more frequently than the ears, making the interaural axis the more stable axis of the two. Rats, on the other hand, rarely pitch past 90°, but they commonly roll over—the ears thus frequently switch directions while the nose does so far more rarely (except via the usual yaw rotations in the locomotor plane).

We come now to why this is relevant to the hairy ball problem. When rats pitch into the inverted posture (a rare maneuver, as we saw) then their head direction cells frequently cease firing or lose their directional selectivity (Calton and Taube, 2005)—this may simply be due to confusion caused by an unfamiliar posture, but it may also be that the attractor network has no mechanism for abruptly switching activity from the (say) North-indicating cells to the South-indicating ones as the rat’s nose tips backward past the vertical. The singularity (the sudden switch in direction) is thus “hidden” in a behavioral place that rats rarely visit. Bats, on the other hand, frequently execute this maneuver but they roll much less often—were they to do so, the activity of the attractor would have to switch direction to accommodate the reversal in ear direction. Again, it seems that the singularity is hidden in a behavioral place the animals rarely inhabit. Thus, the species have evolved a configuration of their respective attractor networks that generally allows free shifting of activity in the planes in which the animals naturally like to move, with the singularities relegated to rotations that are less often executed. This seems like a convenient solution to the hairy ball problem, and one that may even have helped shape the ecological behaviors of these animals.

## Grid Cells

**FIGURE 5:**
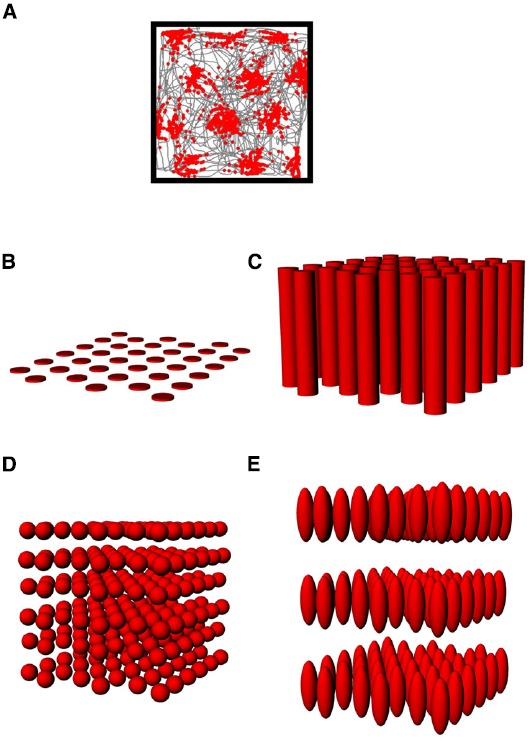
**(A)** The firing pattern of an entorhinal grid cell (same depiction as in Figure 2A), showing the multiple evenly-spaced fields typical of these neurons. **(B–E)** Hypothetical structure of grid fields in 3D space. **(B)** The grid might be flat, with no activity in the vertical space above the floor; **(C)** The fields might be distributed in 3D space but only show spatial structure in the horizontal plane; **(D)** The fields might be symmetric in shape and be distributed throughout the volume in a close-packed array; **(E)** The fields might be distributed volumetrically, but in an anisotropic way (different scale in the vertical dimension).

After the discovery of place cells, both theoretical and experimental studies began to explore the mechanism by which hippocampal neurons are able to self-localize. This theoretical gap was filled by the discovery from the Moser lab (Fyhn et al., 2004; Hafting et al., 2005) of a new class of spatially-modulated neurons in the medial entorhinal cortex (mEC), a major input to the hippocampus. In contrast to place cells, these cells exhibit multiple firing fields that are positioned equidistant to each other (Figure 5A). Because of the striking regularity of this peculiar firing pattern, tessellating all the available surface in a hexagonal grid-like array, the authors named these cells “grid cells.” Importantly, grid cells express their firing patterns in a novel environment and also in the dark, demonstrating an ability to measure distances by using self-motion cues alone (Hafting et al., 2005). This distance-measuring capability confirmed previous intuitions that the spatial encoding implemented by the medial temporal hippocampal system is fundamentally metric—that is, distance- and direction-encoding.

One consequence of the metric encoding revealed by grid cells is that it allows us to ask whether this metricity extends to all three spatial dimensions, or is restricted to the surface on which an animal moves, or even to the horizontal plane (the one orthogonal to gravity). Data on this issue are starting to emerge, although they are still relatively limited. Before looking at these data, we will first review some of the theoretical issues surrounding the extension of a grid into the third dimension (see also the companion data paper by Hayman et al., 2015, for further details on the issues explored here).

### Grid Cells in Three Dimensions—Theoretical Considerations

On a plane, the hexagonal close-packing of firing fields exhibited by grid cells is the most efficient way to pack non-overlapping circles of same size with the least amount of wasted space, and this efficiency and regularity invite speculation that such orderly structure could be used by the spatial system for spatial computations. This notion does not extend completely straightforwardly to the third dimension, however.

As with place cells, there are several hypothetical scenarios by which grid cells encode volumetric space. The cells might show entirely flat encoding, with the grid fields being applied to the floor (Figure 5B); they might extend their fields into the vertical dimension but in a spatially unstructured way, resulting in columnar firing fields (Hayman et al., 2011; Figure 5C) or they might extend the grid into the vertical dimension, thus filling the space with a close-packed array of spherical firing fields, either isotropically (Figure 5D) or anisotropically (Figure 5E).

**FIGURE 6:**
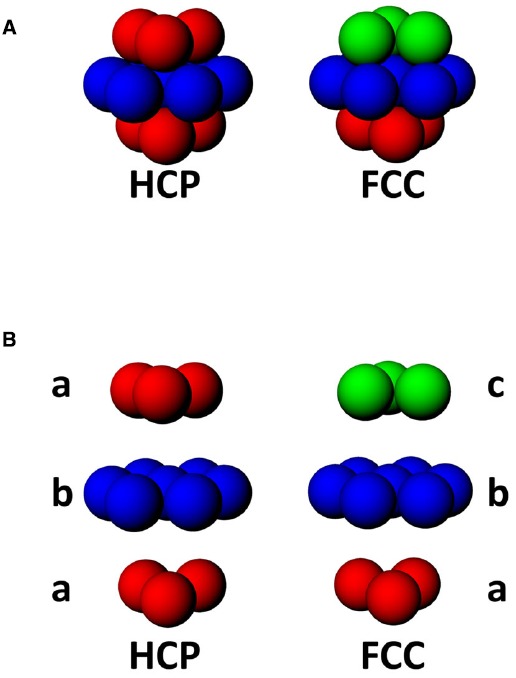
**(A)** Two forms of close-packing of spheres, hexagonal close-packed (HCP) or face-centered cubic (FCC). **(B)** Exploded view of HCP and FCC lattices, showing that HCP involves alternating layers a and b, while FCC comprises triplets of layers, a, b, and c, where c is a 180° horizontal rotation of a.

In a volume, there are two maximally efficient packing arrangements for spheres: hexagonal close packed (HCP) and face-centered-cubic (FCC; Figure 6A; Gauss, 1831). Both HCP and FCC comprise a series of layers of spheres arranged in a hexagonal close-packed planar array, with each layer being offset with respect to the one below. FCC and HCP differ in the amount of offset and thus the sequence of the phases in each layer of spheres (Figure 6B), with HCP having two repeating offsets and FCC having three. These two ways of packing the spheres in a volume are very similar; if one takes a unit of 13 fields, consisting of one central field + 12 surrounding fields, then the difference between FCC and HCP arrangements comprises only 3 fields (Stella and Treves, 2015; see Figure 6).

**FIGURE 7:**
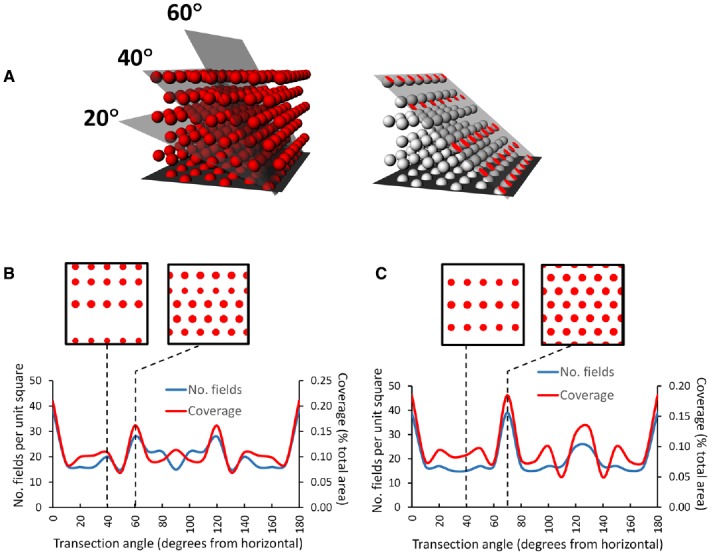
**Pattern of grids seen if a horizontally aligned volumetric close-packed grid lattice, with “fields” of diameter of half the inter-center distance, is transected at different angles by a square planar surface. (A)** A transection square (simulating a sloping surface that a rat might walk on) was aligned with a row of simulated grid fields and then tilted by various increments through the HCP lattice. On the right is the intersection (shown in red) of the tilted plane with some of the grid fields, generating an imaginary grid cell firing pattern which can be analyzed to yield the percentage of the surface covered (“coverage”) and the number of fields. **(B)** HCP lattice: the line graph shows that the transection patterns for both coverage and field number are mirror-symmetric around 90°. Note that in this case, only the horizontal cut results in grid fields with the highest density and greatest rotational symmetry (order 6). The insets show the pattern made when the lattice is cut by a plane tilted at 40° (the tilt used in Hayman et al., 2015) and at 60°, which produces the next densest packing. **(C)** FCC lattice: this is mirror-symmetric around 120°, and produces a close-packed planar transection at 70° as well as horizontal; all the other tilts result in cuts that transect fewer fields, which thus have lower packing density The examples are taken from 40° and 70°. In general, for both lattices a tilted surface tends to cut through fewer fields than a horizontal one (Hayman et al., 2015), raising questions about the potential utility of a grid field lattice for navigation (as compared with a planar grid field that tilts along with the terrain and maintains symmetry and packing density).

An interesting consequence of the HCP closest-packing geometry is that it is inherently anisotropic—the 2D properties of the resulting lattice are different in different orthogonal dimensions. This can be seen in Figures 7A,B, which shows a hypothetical grid-cell grid array following transection of a horizontally aligned hexagonal close-packed HCP 3D lattice at different angles. The figure was made by constructing an HCP lattice of spheres in Rhino 5.0, shrinking them to 50% (the relative size of grid fields with respect to the volume they inhabit) and then transecting with a plane at various angles. The horizontal transection produces a 2D HCP, like a conventional grid cell grid, but transection in the vertical plane produces a different pattern, with sparser coverage of the plane and reduced rotational symmetry.

This observation of fluctuating symmetry at different cutting angles with respect to the horizontal plane has implications for the grid cell encoding of 3D space. If the information contained in grid geometry is used for spatial computation purposes, such computations would be different (less efficient) for the sloped surfaces (except for those angles, such as the 70° slope through an FCC lattice, which transect a plane of fields and hence shows maximum packing density (Figure 7C)) than for horizontal one. The implication is that for an animal walking on a non-horizontal surface, if grid symmetry provides a spatial metric then coding efficiency is greater for a planar grid aligned to the surface than for a volumetric grid lattice aligned to horizontal and transected by the surface. Arguably, then, if the symmetry and density of the grid are important for spatial encoding, then animals that predominantly move on a surface might be better served by a planar grid that follows the surface than a volumetric one anchored to the horizontal plane.

### Grid Cells in Three Dimensions—Data

Data on the issue of grid cell encoding in 3D are scarce: the only relevant published experiments to date are the pegboard experiment of Hayman et al. (2011), mentioned earlier in the context of place cells, and the sloped-arena experiment of Hayman et al. (2015). On the pegboard, consistent with the place cell results, grid fields showed a similar but even more pronounced elongation in the vertical dimension, producing vertical stripes spanning the entire height of the apparatus with a resultant drop in the spatial information content for the vertical dimension only. This is the pattern shown in Figure 5C, in which the cells’ “odometry” (distance-measuring) only functions in the horizontal plane, or perhaps the plane in which the animal is walking. This suggests, as with place cells, that grid cells may encode height with a much coarser resolution than horizontal distance, again implying anisotropy of encoding.

The experiment by Hayman et al. (2015) on the sloped arena aimed to explore whether the grid pattern seen on a slope would resemble that seen on the flat (as if the whole grid plane had tilted together with the surface) or whether it would be consistent with a transection through a close-packed volumetric lattice, as if the sloping surface cut at an angle through a horizontally aligned intrinsic field pattern. Modeling suggested that, assuming grids cells align their (putative) lattices with the horizontal plane, which seems reasonable, then a surface sloped at 40° (which is extremely steep) would intersect fewer grid fields, resulting in a prediction of less firing on the slope than on the flat. In fact, firing was no less, and was if anything slightly increased, with the pattern appearing unchanged with respect to the horizontal. These results were thus, like the place cell results of Knierim and McNaughton (2001) mentioned earlier, consistent with the grid having become tilted with the arena rather than remaining fixed to the horizontal and being transected at 40°. In other words, the results can be explained by having the rats use their plane of locomotion (now tilted at an angle) as their reference frame.

The question of whether grids might form a lattice in a volumetric space will not be fully resolved until recordings are made as animals move through such a space. Meanwhile, the weight of evidence seems to support a planar rather than volumetric coding scheme for spatial neurons in 3D.

## Neural Encoding of Three Dimensional Space—Conclusions and a Hypothesis

The purpose of this article has been to explore, both theoretically and empirically, how the neural encoding of space the properties of which are well characterized in two dimensions, might be structured. A priori, we might suppose that either:

1. The cells form a simple planar representation of heading and distance that are
  a. Referenced to the global space, or
  b. Referenced to the local locomotor surface

Or:

2. The cells form a fully volumetric 3D compass in which heading direction and distance traveled are uniquely specified for any direction the animal faces, in any cardinal plane

The discussion in the preceding sections has explored recent findings concerning the neural representation of space, and has considered some theoretical implications of extending the known representations into the third, vertical dimension. The main conclusion is that introducing an additional dimension fundamentally changes the nature of the encoding problem for neurons encoding metric quantities such as direction and distance. For direction, as we saw earlier, there is a potential problem with singularities that occur when an animal makes certain movements that would require head direction cells to flip their firing directions by 180° —for example in rats pitching from upright to inverted, or for bats rolling from upright to inverted. There is also the problem that if head direction cells are sensitive only to planar rotations, as they appear to be in rats, then an error (i.e., a Berry-Hannay angle) will appear if the animal rotates in planes to which the cells are insensitive.

Distance encoding also encounters problems in 3D. If a distance signal is encoded by the hexagonal close-packed lattice of grid cell firing fields (Stella and Treves, 2015) then in three dimensions the reduced symmetry and packing density of fields would result in a degraded spatial signal. This suggests that the second option, that the cells form a fully volumetric map, may be unlikely, at least in surface-traveling animals like rodents, and possibly also humans.

Is a purely planar map an alternative possibility? This proposition has been raised in several previous discussions (Hayman et al., 2011; Ulanovsky, 2011; Jeffery et al., 2013; Taube and Shinder, 2013), following the report by Hayman et al. (2011) that grid cells did not encode height above ground on a pegboard maze. Planar encoding (for example, referenced only to the plane of the Earth, and disregarding height above ground) could solve some problems, but could cause new ones because if the spatial cells are insensitive to height, or to the ratio of vertical to horizontal distance traveled, then errors in spatial computations might occur—in particular, short-cutting or path integration calculations could be inaccurate. For example, if an animal travels over hilly terrain and takes into account only horizontal distance traveled, then if the legs of its journey occurred on differently graded slopes it may miscalculate the vector need to complete a triangular path back to the start. Conversely, if an animal only computes surface-distance traveled, then for a return journey via a flat (horizontal) route it will overestimate the distance it has to travel to reach home. This latter experiment has actually been done, in an elegant experiment with desert ants by Wohlgemuth et al. (2001); the ants were actually able to compensate for the difference in path length between hilly and flat, albeit crudely (Grah et al., 2007); they thus did not appear to use a purely planar map.

Another problem that could occur with a planar map is that of Berry-Hannay angle errors; as we saw with head direction cells, if the surface curves through three dimensional space then angular errors accumulate which also would degrade navigational computations.

Given these problems, how then can an animal construct a coherent and useful map of three-dimensional space that avoids these pitfalls? One possible compromise solution is for the brains of surface-dwelling animals like rats, and possibly humans, to construct a mosaic of maps that are locally planar but related to each other by their relative distances and directions (Jeffery et al., 2013). By reducing each local part of space to a two-dimensional fragment (a “manifold”), then the problems of interactions between rotations are avoided. By retaining, nonetheless, a systematic relationship between the orientational reference frames (as shown by the way head direction cells maintain consistency between a horizontal and vertical surface (Taube et al., 2013), then an animal can still compute trajectories through space that involve several of these fragments. Whether this can be done is a matter for future behavioral studies.

How might these fragments be stitched together within a larger space? One potential candidate area for this is retrosplenial cortex, which responds to multiple local reference frames (Marchette et al., 2014) and which processes head direction information from both visual and self-motion cues (Chen et al., 1994a,b; Cho and Sharp, 2001) and thus has, potentially, the ability to link these frames in a global super-map. In three dimensions, this would entail modulation of the head direction signal by the angle of the local reference plane—evidence from the Taube lab suggests this indeed happens (Taube et al., 2013).

To summarize, then, the evidence and arguments reviewed here suggest that volumetric encoding by spatial neurons faces computational challenges that might be better solved by reducing the spatial problem to a set of planar ones. This would mean that animals—at least surface-traveling ones—would encode large-scale space in a mosaic fashion rather than as a singular volumetric space. It may even be the case that animals that move freely through volumetric spaces, by flying or swimming, still use planar maps where they can. Comparative studies in both the behavioral and neurophysiology domains will be needed to test this hypothesis and to determine the generality of the encoding processes.

## Author Contributions

All authors discussed the intellectual content of the paper, contributed to its design and contributed text. KJ collated/edited the text and wrote the bulk of the paper.

### Conflict of Interest Statement

The authors declare that the research was conducted in the absence of any commercial or financial relationships that could be construed as a potential conflict of interest.

## References

[B1] Berry M. (1984). Quantal phase factors accompanying adiabatic changes. Proc. R. Soc. A 392, 45–57. 10.1098/rspa.1984.0023

[B2] Berry M. (1988). The geometric phase. Sci. Am. 259, 46–52. 10.1038/scientificamerican1288-46

[B3] Brouwer L. (1912). Über abbildung von mannigfaltigkeiten. Math. Ann. 71, 97–115. 10.1007/BF01456931

[B4] Calton J. L. Taube J. S. (2005). Degradation of head direction cell activity during inverted locomotion. J. Neurosci. 25, 2420–2428. 10.1523/JNEUROSCI.3511-04.2005 15745969 PMC6726092

[B5] Chen L. L. Lin L. H. Barnes C. A. McNaughton B. L. (1994a). Head-direction cells in the rat posterior cortex. II. Contributions of visual and ideothetic information to the directional firing. Exp. Brain Res. 101, 24–34. 10.1007/BF00243213 7843299

[B6] Chen L. L. Lin L. H. Green E. J. Barnes C. A. McNaughton B. L. (1994b). Head-direction cells in the rat posterior cortex. I. Anatomical distribution and behavioral modulation. Exp. Brain Res. 101, 8–23. 10.1007/BF00243212 7843305

[B7] Cho J. Sharp P. E. (2001). Head direction, place, and movement correlates for cells in the rat retrosplenial cortex. Behav. Neurosci. 115, 3–25. 10.1037/0735-7044.115.1.3 11256450

[B8] Finkelstein A. Derdikman D. Rubin A. Foerster J. N. Las L. Ulanovsky N. (2015). Three-dimensional head-direction coding in the bat brain. Nature 517, 159–164. 10.1038/nature14031 25470055

[B8a] Fyhn M. Molden S. Witter M. P. Moser E. I. Moser M. B. (2004). Spatial representation in the entorhinal cortex. Science 305, 1258–1264. 15333832 10.1126/science.1099901

[B9] Gauss C. F. (1831). Besprechung des Buchs von L. A. Seeber: intersuchungen über die eigenschaften der positiven ternären quadratischen Formen usw. Göttingsche Gelehrte Anzeigen 2, 188–196.

[B10] Gibson B. Butler W. N. Taube J. S. (2013). The head-direction signal is critical for navigation requiring a cognitive map but not for learning a spatial habit. Curr. Biol. 23, 1536–1540. 10.1016/j.cub.2013.06.030 23891111 PMC4106916

[B11] Goodridge J. P. Dudchenko P. A. Worboys K. A. Golob E. J. Taube J. S. (1998). Cue control and head direction cells. Behav. Neurosci. 112, 749–761. 10.1037/0735-7044.112.4.749 9733184

[B12] Grah G. Wehner R. Ronacher B. (2007). Desert ants do not acquire and use a three-dimensional global vector. Front. Zool 4:12. 10.1186/1742-9994-4-12 17475021 PMC1868725

[B12a] Hafting T. Fyhn M. Molden S. Moser M. B. Moser E. I. (2005). Microstructure of a spatial map in the entorhinal cortex. Nature 436, 801–806. 15965463 10.1038/nature03721

[B13] Hannay J. H. (1985). Angle variable holonomy in adiabatic excursion of an integrable Hamiltonian. J. Phys. A Math. Gen. 18, 221–230. 10.1088/0305-4470/18/2/011

[B14] Hayman R. M. Casali G. Wilson J. J. Jeffery K. J. (2015). Grid cells on steeply sloping terrain: evidence for planar rather than volumetric encoding. Front. Psychol. 6:925 10.3389/fpsyg.2015.00925 PMC4502341 26236245

[B15] Hayman R. Verriotis M. A. Jovalekic A. Fenton A. A. Jeffery K. J. (2011). Anisotropic encoding of three-dimensional space by place cells and grid cells. Nat. Neurosci. 14, 1182–1188. 10.1038/nn.2892 21822271 PMC3166852

[B16] Jeffery K. J. (2007). Integration of the sensory inputs to place cells: what, where, why, and how? Hippocampus 17, 775–785. 10.1002/hipo.20322 17615579

[B17] Jeffery K. J. Jovalekic A. Verriotis M. Hayman R. (2013). Navigating in a three-dimensional world. Behav. Brain Sci. 36, 523–543. 10.1017/S0140525X12002476 24103594

[B18] Knierim J. J. McNaughton B. L. (2001). Hippocampal place-cell firing during movement in three-dimensional space. J. Neurophysiol. 85, 105–116. 11152711 10.1152/jn.2001.85.1.105

[B19] Knierim J. J. McNaughton B. L. Poe G. R. (2000). Three-dimensional spatial selectivity of hippocampal neurons during space flight. Nat. Neurosci. 3, 209–210. 10.1038/72910 10700250

[B20] Marchette S. A. Vass L. K. Ryan J. Epstein R. A. (2014). Anchoring the neural compass: coding of local spatial reference frames in human medial parietal lobe. Nat. Neurosci. 17, 1598–1606. 10.1038/nn.3834 25282616 PMC4309016

[B21] Marozzi E. Jeffery K. J. (2012). Place, space and memory cells. Curr. Biol. 22:R939–R942. 10.1016/j.cub.2012.10.022 23174291

[B22] Moser E. I. Kropff E. Moser M. B. (2008). Place cells, grid cells, and the brain’s spatial representation system. Annu. Rev. Neurosci. 31, 69–89. 10.1146/annurev.neuro.31.061307.090723 18284371

[B23] O’Keefe J. Burgess N. (1996). Geometric determinants of the place fields of hippocampal neurons. Nature 381, 425–428. 10.1038/381425a0 8632799

[B24] O’Keefe J. Dostrovsky J. (1971). The hippocampus as a spatial map. Preliminary evidence from unit activity in the freely-moving rat. Brain Res. 34, 171–175. 10.1016/0006-8993(71)90358-1 5124915

[B25] Ranck J. J. (1984). Head-direction cells in the deep cell layers of dorsal presubiculum in freely moving rats. Soc. Neurosci. Abstr. 10, 599.

[B26] Sharp P. E. Blair H. T. Cho J. (2001). The anatomical and computational basis of the rat head-direction cell signal. Trends Neurosci. 24, 289–294. 10.1016/S0166-2236(00)01797-5 11311382

[B27] Skaggs W. E. Knierim J. J. Kudrimoti H. S. McNaughton B. L. (1995). A model of the neural basis of the rat’s sense of direction. Adv. Neural Inf. Process. Syst. 7, 173–180. 11539168

[B28] Stackman R. W. Tullman M. L. Taube J. S. (2000). Maintenance of rat head direction cell firing during locomotion in the vertical plane. J. Neurophysiol. 83, 393–405. 10634882 10.1152/jn.2000.83.1.393

[B28a] Stella F. Treves A. (2015). The self-organization of grid cells in 3D. eLife 4. 10.7554/eLife.05913 25821989 PMC4453437

[B29] Taube J. S. (2007). The head direction signal: origins and sensory-motor integration. Annu. Rev. Neurosci. 30, 181–207. 10.1146/annurev.neuro.29.051605.112854 17341158

[B30] Taube J. S. (2011). Head direction cell firing properties and behavioural performance in 3-D space. J. Physiol. 589, 835–841. 10.1113/jphysiol.2010.194266 20855436 PMC3060363

[B31] Taube J. S. Muller R. U. Ranck J. B. J. (1990a). Head-direction cells recorded from the postsubiculum in freely moving rats. I. Description and quantitative analysis. J. Neurosci. 10, 420–435. 2303851 10.1523/JNEUROSCI.10-02-00420.1990 PMC6570151

[B32] Taube J. S. Muller R. U. Ranck J. B. J. (1990b). Head-direction cells recorded from the postsubiculum in freely moving rats. II. Effects of environmental manipulations. J. Neurosci. 10, 436–447. 2303852 10.1523/JNEUROSCI.10-02-00436.1990 PMC6570161

[B33] Taube J. S. Shinder M. (2013). On the nature of three-dimensional encoding in the cognitive map: commentary on Hayman, Verriotis, Jovalekic, Fenton, and Jeffery. Hippocampus 23, 14–21. 10.1002/hipo.22074 22996337 PMC3526945

[B34] Taube J. S. Wang S. S. Kim S. Y. Frohardt R. J. (2013). Updating of the spatial reference frame of head direction cells in response to locomotion in the vertical plane. J. Neurophysiol. 109, 873–888. 10.1152/jn.00239.2012 23114216 PMC3567391

[B35] Ulanovsky N. (2011). Neuroscience: how is three-dimensional space encoded in the brain? Curr. Biol. 21:R886–R888. 10.1016/j.cub.2011.09.031 22075427

[B36] Valerio S. Clark B. J. Chan J. H. Frost C. P. Harris M. J. Taube J. S. (2010). Directional learning, but no spatial mapping by rats performing a navigational task in an inverted orientation. Neurobiol. Learn. Mem. 93, 495–505. 10.1016/j.nlm.2010.01.007 20109566 PMC2862784

[B37] Wohlgemuth S. Ronacher B. Wehner R. (2001). Ant odometry in the third dimension. Nature 411, 795–798. 10.1038/35081069 11459057

[B38] Yartsev M. M. Ulanovsky N. (2013). Representation of three-dimensional space in the hippocampus of flying bats. Science 340, 367–372. 10.1126/science.1235338 23599496

[B39] Zhang K. (1996). Representation of spatial orientation by the intrinsic dynamics of the head-direction cell ensemble: a theory. J. Neurosci. 16, 2112–2126. 8604055 10.1523/JNEUROSCI.16-06-02112.1996 PMC6578512

